# PRISM: A Platform for Imaging in Precision Medicine

**DOI:** 10.1200/CCI.20.00001

**Published:** 2020-06-01

**Authors:** Ashish Sharma, Lawrence Tarbox, Tahsin Kurc, Jonathan Bona, Kirk Smith, Pradeeban Kathiravelu, Erich Bremer, Joel H. Saltz, Fred Prior

**Affiliations:** ^1^ Emory University School of Medicine, Atlanta, GA; ^2^ University of Arkansas for Medical Sciences, Little Rock, AR; ^3^ Stony Brook University, Stony Brook, NY

## Abstract

**PURPOSE:**

Precision medicine requires an understanding of individual variability, which can only be acquired from large data collections such as those supported by the Cancer Imaging Archive (TCIA). We have undertaken a program to extend the types of data TCIA can support. This, in turn, will enable TCIA to play a key role in precision medicine research by collecting and disseminating high-quality, state-of-the-art, quantitative imaging data that meet the evolving needs of the cancer research community

**METHODS:**

A modular technology platform is presented that would allow existing data resources, such as TCIA, to evolve into a comprehensive data resource that meets the needs of users engaged in translational research for imaging-based precision medicine. This Platform for Imaging in Precision Medicine (PRISM) helps streamline the deployment and improve TCIA’s efficiency and sustainability. More importantly, its inherent modular architecture facilitates a piecemeal adoption by other data repositories.

**RESULTS:**

PRISM includes services for managing radiology and pathology images and features and associated clinical data. A semantic layer is being built to help users explore diverse collections and pool data sets to create specialized cohorts. PRISM includes tools for image curation and de-identification. It includes image visualization and feature exploration tools. The entire platform is distributed as a series of containerized microservices with representational state transfer interfaces.

**CONCLUSION:**

PRISM is helping modernize, scale, and sustain the technology stack that powers TCIA. Repositories can take advantage of individual PRISM services such as de-identification and quality control. PRISM is helping scale image informatics for cancer research at a time when the size, complexity, and demands to integrate image data with other precision medicine data-intensive commons are mounting.

## INTRODUCTION

The Precision Medicine Initiative in Oncology is envisioned to “encourage and support . . . new approaches for detecting, measuring, and analyzing a wide range of biomedical information—including molecular, genomic, cellular, clinical, behavioral, physiological, and environmental parameters.”^1(p794)^ Precision medicine requires the ability to classify patients into specialized cohorts that differ in their susceptibility to a particular disease, in the biology of the disease, response to therapy,^2^ and so on. Imaging data and, in particular, quantitative imaging features have been identified as a critical source of information when creating such cohorts for precision oncology. Radiomics and pathomics, where quantitative features are extracted from radiology^3-5^ and digital pathology,^6,7^ provide valuable diagnostic and prognostic indicators of cancer.^8-13^ Identifying such quantitative imaging phenotypes across scale through the use of radiomics, deep learning, and so on also provides an alternative approach to improve our understanding of cancer biology.^14,15^ However, these methodologies of leveraging quantitative imaging for clinical and basic research require large collections of well-curated diverse data sets for reproducible development and validation.

**CONTEXT**

**Key Objective**
Open access information repositories advance cancer research by enabling the creation of new study cohorts and reuse of data to address new research questions. The Cancer Image Archive has served as the National Cancer Institute’s open image repository for the past decade, and through the Platform for Imaging in Precision Medicine project its technology base and capabilities are being greatly enhanced.
**Knowledge Generated**
Advanced research into imaging phenotypes and quantitative image analyses in both radiology and pathology are generating a new type of data: image-derived feature sets. The tools for semantic integration of clinical and quantitative image data across scale we are developing will enable new research directions and support advanced machine learning algorithm development.
**Relevance**
Quantitative imaging and omics data (eg, radiogenomics) are proving to be essential new tools to advance our understanding of cancer mechanisms and improve our ability to diagnose and track response to cancer therapy. State-of-the-art, open-access information repositories are essential to enable these techniques to produce actionable clinical knowledge.

Although a growing number of cancer imaging and precision medicine information resources are coming on line,^16-18^ the Cancer Imaging Archive (TCIA) has been the primary resource of the National Cancer Institute (NCI) for acquiring, curating, managing, and distributing images and related data to support cancer research since its creation in 2011. TCIA radiology and pathology images are collected from > 46,500 human subjects as well as associated clinical data, image-derived features, and annotations.^19^ TCIA also manages a growing number of preclinical image collections, including patient-derived xenograft models. It is visited by approximately 20,000 users per month from approximately 130 countries, exports > 1 PB of data per year and has provided data to > 900 peer-reviewed publications and graduate theses. It is the primary image repository for several NCI programs,^20-24^ clinical trials,^25^ and various challenges.^20,26-30^

Even though TCIA has been highly successful, it has some inherent challenges that limit its ability to support the growing field of precision oncology and data sciences. These challenges are not only inherent to TCIA but also observed in institutional data repositories and other large data-sharing activities. In response to these challenges, in 2017 we began work on the Platform for Imaging in Precision Medicine (PRISM). This article summarizes our ongoing developments in PRISM, in particular: novel solutions for managing radiomics and pathomics data sets, managing and integrating clinical data sets, supporting semantic search to ease data discovery, and evolving the curation pipelines to improve throughput. Finally, although TCIA remains the primary driver of PRISM, one of the primary objectives of PRISM is also to modernize and modularize the underlying technology stack so that individual components can be adopted piecemeal.

## CHALLENGES

The design and development of PRISM stem from the core premise that well-curated data repositories, with semantically linked collections that permit researchers to integrate information across scale, are essential to cancer imaging and precision medicine research. Simply archiving images is no longer sufficient in today’s precision medicine approach to cancer treatment. Researchers have identified the need to analyze integrated data sets consisting of tightly coupled radiology and pathology images with clinical context and features extracted from the images. Through a variety of discussions, TCIA feature requests, surveys, and so on, the following challenges were identified. These challenges have been instrumental in guiding and prioritizing the design and development of PRISM:

- Comprehensive data management and curation to include clinical data, a full range of imaging modalities, pathology images, and radiomic and pathomic features.
- Better tools for curating high-quality data sets at large scales.
- Integration across clinical, radiology, pathology images, and derived feature sets to support queries involving interrelationships between clinical course, response to treatment, and the acquired images and computed features.
- Semantic search that links images, clinical data, and derived features and helps in data discovery and interoperability.
- Tools to encourage data sharing and promote reproducible research.
- A modular architecture that allows piecemeal adoption of capabilities as well as a near-seamless ability to move between cloud and an on-premise deployment.

## PRISM

**FIG 1.**
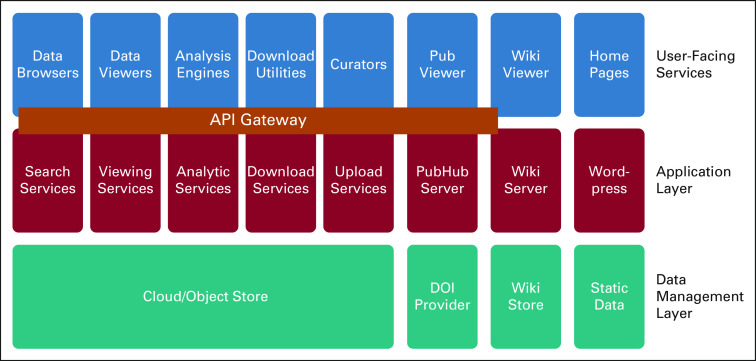
A high-level Platform for Imaging in Precision Medicine (PRISM) architecture diagram illustrating the key functional microservices, a representational state transfer interface, and an underlying object store where all raw data are stored. A set of cross-cutting security services are available for user authentication and access control. API, application programming interface; DOI, Digital Object Identifier.

PRISM is taking a systematic approach to address these challenges via a new architectural framework that builds on the principles of microservice architecture and a rich ecosystem of application programming interfaces (APIs). As illustrated in Figure 1, it targets a better modularization of existing software and more efficient incorporation of new services, extensibility, and scalability.

Applications in the top layer may use any of the underlying services to accomplish a task. Multiple applications may perform similar functions but targeted to different user communities. All functions in the top two layers are interconnected by APIs. In the PRISM architecture, we have chosen to enhance this framework with an API Gateway,^31^ which can also deal with user authentication for services. The middle layer includes server-side functions supported by databases and Resource Description Framework triple stores^32^ and accessed via the API gateway (except landing pages, wiki, and service desk). Finally, the bottom layer comprises the object stores and external services. The PRISM architecture is explicitly designed to manage data housed in an object store and accessed by standard interfaces such as S3 and OpenStack Object Storage.^33^

### Image and Feature Management

The design and development of PRISM are driven to support “image-omic studies,” a research design that involves the integration of clinical data, imaging data, quantitative features extracted from the images, and molecular data. Such studies enable a highly data- driven approach to diagnosis and outcome prediction^34^ and are a key component of precision medicine. Indeed, many research groups have developed methods for linked characterizations of imaging features, clinical outcome, and omics signatures and studied their relevance in clinical research.^6-12,35-47^

**FIG 2.**
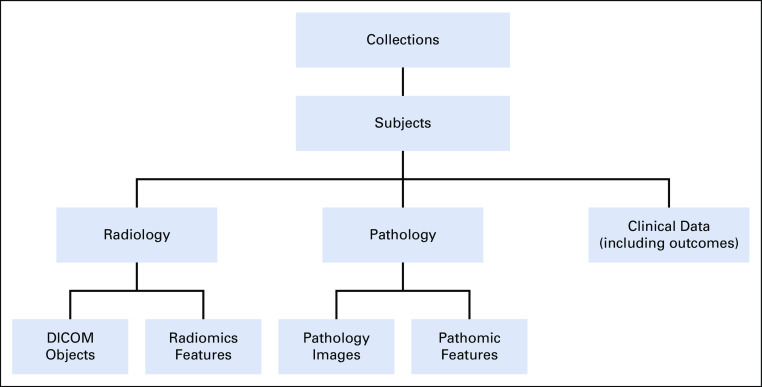
Simplified data model illustrating the linkages between various data types and the organization of data as collections. Subjects are identified consistently within a collection, and all data for that subject are properly linked. Because data are de-identified before leaving the submitting site, it is possible for the same subject to appear in multiple collections, with different subject identifiers. Because the collections have limited overlap, this probability is considered to be low. DICOM, Digital Imaging and Communications in Medicine.

Locating and accessing data cohorts with the relevant information requires that besides imaging metadata, any associated clinical and demographic data be indexed and part of the data query process. Although it would be desirable to index and search across imaging features, it becomes difficult to harmonize features and make them part of the query process. It is much easier to index the availability of features and their provenance, so users can make that information part of the query process. However, imaging features must be part of a data cohort. To maintain linkages across the various data types and manage the data across multiple collections, PRISM builds on the TCIA data model, as shown in Figure 2.

#### Image data management.

The PRISM data model organizes data as collections. A collection typically includes studies from several subjects (patients), and each subject has data of multiple data types, such as radiology and pathology images, radiomic and pathomic features, and clinical data. Radiology image data are represented as Digital Imaging and Communications in Medicine (DICOM) objects and are managed using the open-source National Biomedical Imaging Archive (NBIA) software package.^48^ NBIA functions as an application layer that sits over a MySQL relational database. PRISM is expanding the radiology data management capabilities and adding support for the new DICOMweb^49^ representational state transfer (REST or RESTful) APIs. The use of such standardized APIs will allow the adoption of off-the-shelf DICOM viewers and directly query and retrieve DICOM data.

Unlike radiology, there are no common standards for pathology image data. Therefore, PRISM includes PathDB, a pathology data management system that manages and organizes whole slide images and pathomic features and the provenance of the features. Included with PathDB is a web application called FeatureMap. FeatureMap allows users to view and interact with feature maps. A feature map is a composite representation in the form of a low-resolution image of one or more classification probability maps; probability maps are generated on whole-slide tissue images by deep learning methods.^50^

Access control for image data and associated nonimaging data, such as features and any available clinical data, are managed at the collection level. If a user has access to a particular collection, then all data under that collection are also made available. User access information is managed in a Lightweight Directory Access Protocol server, though plans are underway to migrate the user authentication and authorization information to an open-source system called Keycloak^51^ that uses modern security standards.

#### Radiomics and pathomics features.

FeatureBase is responsible for storing and indexing large volumes of imaging features so that user-facing query and visualization applications can efficiently interact with them. Pathomic features can include individual segmented nuclei/cells and their morphology as well as features indicating patterns and the likelihood of macro structures, such as lymphocyte patterns, or characterization of tumoral and stromal regions. Pathomics can become very large. For example, segmenting nuclei in a data set of 1,000 images can easily generate more than a billion segmented objects and tens of billions of imaging features. To address the complexity and scale of pathomics data, PRISM has adapted the FeatureDB service of QuIP^52^ to implement FeatureBase.

Although FeatureBase was developed to support pathomics features, there is a significant overlap between the 2 data types and how researchers interact with features. FeatureBase can index individual objects and store them as polygons, whereas features computed for segmented objects are stored as feature vectors, spatial patterns, or probability maps. A probability map partitions an image into a uniform mesh of image patches. Each image patch is assigned a probability value (by a machine/deep learning method), which indicates the probability of the image patch belonging to a class (eg, grade 3 tumor). For pathomic features, the various imaging features are represented as GeoJSON-compliant JSON documents that are then managed and indexed in a MongoDB database. Unlike pathology, in radiology, the DICOM community has standards for representing segmentations and probability maps, as well as structured representations of computed features. Therefore, instead of using GeoJSON, we are adopting DICOM standards for representing radiomic features but indexing and managing them in MongoDB. The use of a shared environment for radiomic and pathomic features is expected to improve linkages between radiomics and pathomics data for integrated exploration and analysis.

## MAKING THE DATA FAIR

The stewardship of image data needs to adhere to the FAIR (findable, accessible, interoperable, reusable) principles,^53^ to achieve its full potential as a scientific resource. This is a key design tenet of PRISM. PRISM-based resources have to be agile to meet the changing needs and technologies that are in use by the community, such as the increasing reliance on REST APIs and advanced computational statistics engines to support programmatic interoperability at scale. In particular, data assets produced and consumed by image analysis need to be available as components of an “API ecosystem” as part of the overarching normalization of Research Data Commons.^54^ The fluid nature of these new software engineering environments comes with its own challenges, such as the need for continuous API design and distributed authorization.^55^

### Findable: Semantic Integration and Search

Semantic integration in PRISM aims to make image collections and associated nonimage data more findable, accessible, interoperable, and reusable. Our approach goes beyond the specific need to make data findable by also addressing the underlying challenge of integrating and managing diverse nonimage data associated with image collections. PRISM integrates and manages nonimage data using ontology-based representation patterns that account for explicit and implicit connections among the data across the source data sets.^56^ Instances in the data are linked to ontology classes that define and represent the entities that the data are about (eg, anatomic locations, disease types, diagnosis). The Open Biomedical Ontologies (OBO) Foundry^57^ is a collection of axiomatically rich ontologies adhering to common design principles and using a consistent shared representational strategy based on Basic Formal Ontology^58^ to achieve interoperability across subject areas. OBO ontologies are available for reuse under a permissive license (CC BY 4.0). PRISM uses many OBO resources, including the Human Disease Ontology,^59^ the Ontology for Biomedical Investigations,^60^ and the Uber Anatomy Ontology (Uberon).^61^

**FIG 3.**
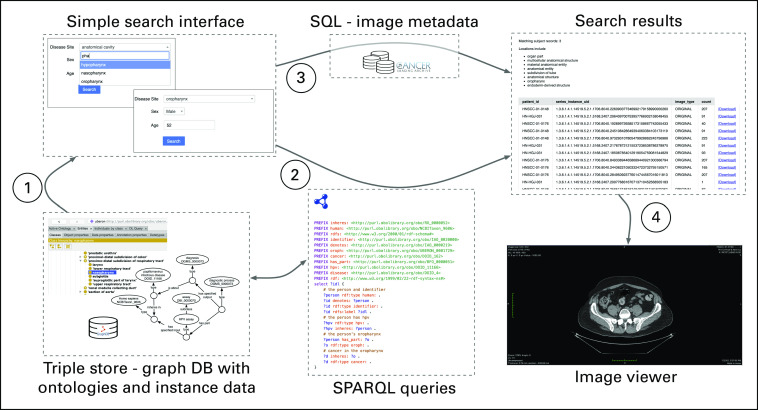
Ontology-driven semantic search utilizing both structured query language (SQL) and SPARQL queries against Platform for Imaging in Precision Medicine (PRISM) data management components. DB, database; SPARQL, SPARQL Protocol and RDF query language.

Work is ongoing to develop ontology-driven semantic search tools that make use of the representations underlying our semantic integration efforts. Richer user-facing tools for search and exploration of nonimage data in image collections will allow queries across collections that combine demographics, tumor location, disease types, and other similar data. We have developed a proof-of-concept query interface that allows users to identify records matching criteria on the basis of fields in nonimage data that were previously not queryable—for instance, finding records across head and neck cancer collections for male patients > 55 years of age with a positive HPV diagnosis and a primary tumor in the oropharynx. Figure 3 illustrates the ontology-driven semantic search strategy, in which a simple search interface populated using ontologies and linked instances generates SPARQL queries to search the ontology-linked nonimage data (stored in a triple-store database), as well as structured query language (SQL) queries for image metadata stored in a relational database. The results link directly to downloadable/viewable images from matching records. ARIES (Arkansas Image Enterprise System),^62^ a PRISM instance hosting neuroimaging data for University of Arkansas for Medical Sciences researchers, provides an early testbed to deploy and refine the PRISM approach to semantic integration.

### Accessible: Visualization and Data Exploration Apps

PRISM includes a variety of user-facing web applications that allow researchers to explore a repository and create and examine cohorts. Web applications enabled by the modern browser have the advantage of being assembled in the browser’s sandbox, which comes with significant advantages when operating cloud resources safely. Such web applications, often described as progressive web apps, are an ideal environment to engage PRISM’s APIs to drive the various web viewers and data exploration tools.

**FIG 4.**
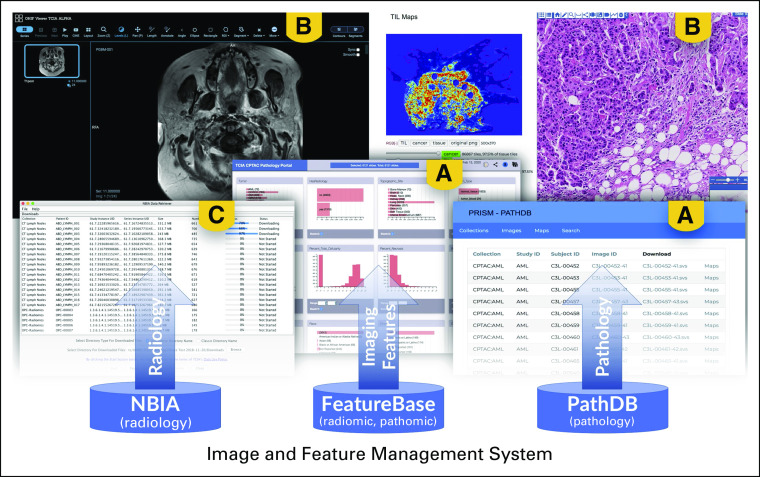
The various data management systems interfacing with applications for (A) data exploration, (B) visualization, and (C) bulk download. (A) DataScope graphical analytics data exploration tools and the PathDB query interface (an equivalent interface exists for radiology images). (B) Left image is the Open Health Imaging Foundation image viewer for radiology image visualization, and the right image is the caMicroscope pathology image viewer. (C) One of the download mechanisms based on a shopping cart model. NBIA, National Biomedical Imaging Archive.

PRISM now includes the Open Health Imaging Foundation viewer^63^ for visualizing radiology objects and the caMicroscope viewer^50,52^ for visualizing digital pathology images. These viewers interface with the respective image management systems (Fig 4). A high-speed bulk download mechanism is available to help users reliably download large amounts of radiology data. A similar mechanism to support the download of pathology data is under development. For interactive data exploration, a suite of task-specific data portals, such as the Clinical Proteomic Tumor Analysis Consortium Pathology Portal,^64^ have been built using a declarative visualization tool called DataScope.^65^ These provide the foundation for a series of generic data exploration environments that are being built and will be released in the coming months as part of the PRISM tech stack. Finally, the accessibility to PRISM-managed data, via APIs, has allowed third parties to develop integrations with research frameworks such as BioConductor,^66^ third-party applications such as 3DSlicer,^67^ and data science environments such as Jupyter notebooks.^68^

### Interoperable: Data Curation

Careful curation and strict quality-control processes have been instrumental activities that have led to the success of TCIA. PRISM builds on the TCIA experience and includes tools that are capable of curating diverse data sets at large scales. The modular design of PRISM allows us to disseminate these capabilities and make them available as stand-alone modules that can be used as drivers of individual research imaging repositories. This includes dissemination of knowledge to the wider research community in areas of DICOM de-identification^69^ and open data.^70^

PRISM is adopting and modernizing the suite of advanced tools, procedures, and scalable workflows for semi-automated data curation, quality control, and enhancement, which have allowed the repository to continuously grow. Data curation in PRISM uses the Posda tool suite^71^ to implement its curation workflows. Posda is a set of curation workflow tools developed to provide a mechanism to ensure the scientific utility of data and to eliminate protected health information as well as improving the scalability of curation workflow. Posda supports a single curation pipeline dealing with all object types defined by the DICOM standard (images, radiation therapy objects, structured reports, segmentation, and so on). This pipeline performs integrity checks automatically on a bulk basis, applies revisions to data sets, tracks all changes in a revision tracker permitting rollback if needed, and rapidly identifies potential duplicate data sets on the basis of stored hash codes, without identifying the individual.

PRISM is extending Posda with new workflows to support pathology and pathomic features. Curation tools are being interfaced with semantic integration and ontology toolkits as new Posda pipelines and curation procedures. The overarching objective of curation is to ensure compliance governing disclosure of protected health information and ensure that data formats are reusable and have enough semantic metadata so that researchers can unambiguously find the data they need.

### Reusable: Digital Object Identifiers

To incentivize data sharing and promote research reproducibility, many publishers now encourage authors to provide data citations. PRISM leverages the popular Digital Object Identifier (DOI) management system called DataVerse^72^ for “publishing” user-generated results and issuing and managing DOIs. DOIs are well-recognized mechanisms to make the provided data unique, persistent, and citable.^73^ DataVerse is being integrated with FeatureBase to better support image-omic features and the various other data-management systems. The metadata schema used by DataVerse allows PRISM to include attributes that facilitate versioning and others that capture the relationships between the data set being registered and related publications/data sets.

## OPERATING AT SCALE: THE PRISM TECH STACK

TCIA was originally implemented as a collection of mirrored and load-balanced virtual machines (VMs) and shared bulk storage for all of the VMs. This has allowed TCIA to maintain a 99.5% uptime. The main headache with using VMs is that the collection of systems making up TCIA is difficult to deploy and requires intimate knowledge of the interconnections between systems to keep TCIA updated and running. More importantly, the tech stack is tightly coupled, and this makes it difficult to distribute and adopt piecemeal individual capabilities.

In PRISM, the tech stack is being modularized and driven as a set of RESTful web services, including data services, that interface with data stored on a modern object storage system. These services are accessed via APIs that are made available through a centralized API gateway. Additional core services, such as load balancers and centralized security services, are also made available. PRISM will rely on Kubernetes,^74^ an orchestrated container management environment where the interconnections and interfaces between containers making up subsystems, as well as the interconnections between subsystems, are automatically configured using scripts. This simplifies deployment and maintenance of PRISM-based sites regardless of whether the sites are hosted locally on dedicated hardware or in virtualized or cloud-based environments.

All PRISM components developed by our team are released open source under the BSD 3-Clause “New” or “Revised” License or the Apache 2.0 License. Available examples include the Posda curation toolkit,^75^ the QuIP Pathology and pathomics management services,^76^ and the caMicroscope pathology viewer.^77^ Additional modules are similarly distributed. Components such as the Kubernetes orchestration software and API gateway^78^ are open-source tools developed by others.

In conclusion, realizing the promise of precision medicine in enabling better treatment strategies for cancer, a complex multifactorial disease state, will largely depend on how well we synthesize information across multiple scales from the patient down to the molecular level. Today, treatment strategies are often developed by gleaning information through qualitative and subjective interpretations of images combined with molecular characterizations and clinical data. Although molecular characterizations inform prognosis and targeted therapy decisions, image information is a crucial component in the overall decision-making process. Radiomics and pathomics studies provide highly detailed, quantitative, and reproducible descriptions and characterizations of tumor structure and function at complementary biologic scales. The complexity and sizes of primary and derived data sets in radiomics and pathomics dictate scalable and extensible software infrastructures to curate, manage, and share said data sets. PRISM provides capabilities that allow researchers to address these issues of data management and integration, thus allowing them to quantitatively incorporate imaging data. These capabilities will enable the cancer research community to synthesize information across multiple scales, a key tenet of precision medicine for cancer.

Consider a research team studying lung cancer. A PRISM-based repository will allow the team to use semantic query capabilities to pool data from multiple collections to create the requisite cohort of, say, patients with lung adenocarcinoma, with linkages across various images, features, feature provenance, and molecular characteristics. The research team can manage, explore, and refine results from their analyses within their collaboration. They will be able to upload their analysis results and images to the community PRISM instance if they would like to share them with the research community at the completion of their study.

## References

[1] Collins FS Varmus H A new initiative on precision medicine N Engl J Med 372 793 795 2015 25635347 10.1056/NEJMp1500523 PMC5101938

[2] National Research Council: Toward precision medicine: Building a knowledge network for biomedical research and a new taxonomy of disease. Washington, DC, National Academies Press, 2011 22536618

[3] Bi WL Hosny A Schabath MB et al Artificial intelligence in cancer imaging: Clinical challenges and applications CA Cancer J Clin 69 127 157 2019 30720861 10.3322/caac.21552 PMC6403009

[4] Hosny A Parmar C Quackenbush J et al Artificial intelligence in radiology Nat Rev Cancer 18 500 510 2018 29777175 10.1038/s41568-018-0016-5 PMC6268174

[5] Yankeelov TE Mankoff DA Schwartz LH et al Quantitative imaging in cancer clinical trials Clin Cancer Res 22 284 290 2016 26773162 10.1158/1078-0432.CCR-14-3336 PMC4717912

[6] Cooper LA Kong J Gutman DA et al An integrative approach for in silico glioma research IEEE Trans Biomed Eng 57 2617 2621 2010 20656651 10.1109/TBME.2010.2060338 PMC3289150

[7] Cooper LA Kong J Gutman DA et al Integrated morphologic analysis for the identification and characterization of disease subtypes J Am Med Inform Assoc 19 317 323 2012 22278382 10.1136/amiajnl-2011-000700 PMC3277636

[8] Aerts HJ, Velazquez ER, Leijenaar RT, et al: Decoding tumour phenotype by noninvasive imaging using a quantitative radiomics approach. Nat Commun 5:4006, 2014 [Erratum: Nat Commun 5:4644, 2014] 10.1038/ncomms5006 PMC4059926 24892406

[9] Parmar C Leijenaar RT Grossmann P et al Radiomic feature clusters and prognostic signatures specific for lung and head & neck cancer Sci Rep 5 11044 2015 26251068 10.1038/srep11044 PMC4937496

[10] Parmar C Rios Velazquez E Leijenaar R et al Robust RADIOMICS feature quantification using semiautomatic volumetric segmentation PLoS One 9 e102107 2014 25025374 10.1371/journal.pone.0102107 PMC4098900

[11] Kumar V Gu Y Basu S et al Radiomics: The process and the challenges Magn Reson Imaging 30 1234 1248 2012 22898692 10.1016/j.mri.2012.06.010 PMC3563280

[12] Lambin P Rios-Velazquez E Leijenaar R et al Radiomics: Extracting more information from medical images using advanced feature analysis Eur J Cancer 48 441 446 2012 22257792 10.1016/j.ejca.2011.11.036 PMC4533986

[13] Gillies RJ Kinahan PE Hricak H Radiomics: Images are more than pictures, they are data Radiology 278 563 577 2016 26579733 10.1148/radiol.2015151169 PMC4734157

[14] Thrall JH Personalized medicine Radiology 231 613 616 2004 15163802 10.1148/radiol.2313040323

[15] Thrall JH Trends and developments shaping the future of diagnostic medical imaging: 2015 Annual Oration in Diagnostic Radiology Radiology 279 660 666 2016 27183401 10.1148/radiol.2016160293

[16] Deist TM Jochems A van Soest J et al Infrastructure and distributed learning methodology for privacy-preserving multi-centric rapid learning health care: euroCAT Clin Transl Radiat Oncol 4 24 31 2017 29594204 10.1016/j.ctro.2016.12.004 PMC5833935

[17] Lopez MG, Posada N, Moura DC, et al: BCDR: A breast cancer digital repository. Presented at the 15th International Conference on Experimental Mechanics, Porto, Portugal, July 22-27, 2012

[18] Bhuvaneshwar K Belouali A Singh V et al G-DOC Plus—An integrative bioinformatics platform for precision medicine BMC Bioinformatics 17 193 2016 27130330 10.1186/s12859-016-1010-0 PMC4851789

[19] Prior F Smith K Sharma A et al The public cancer radiology imaging collections of The Cancer Imaging Archive Sci Data 4 170124 2017 28925987 10.1038/sdata.2017.124 PMC5827108

[20] Kalpathy-Cramer J Freymann JB Kirby JS et al Quantitative Imaging Network: Data sharing and competitive algorithm validation leveraging The Cancer Imaging Archive Transl Oncol 7 147 152 2014 24772218 10.1593/tlo.13862 PMC3998686

[21] Armato SG III Hadjiiski L Tourassi GD et al LUNGx Challenge for computerized lung nodule classification: Reflections and lessons learned J Med Imaging (Bellingham) 2 020103 2015 26158094 10.1117/1.JMI.2.2.020103 PMC4489507

[22] Zheleznyak A Shokeen M Achilefu S Nanotherapeutics for multiple myeloma Wiley Interdiscip Rev Nanomed Nanobiotechnol 10 e1526 2018 29701006 10.1002/wnan.1526 PMC6185771

[23] Clarke LP Nordstrom RJ Zhang H et al The Quantitative Imaging Network: NCI’s historical perspective and planned goals Transl Oncol 7 1 4 2014 24772201 10.1593/tlo.13832 PMC3998696

[24] Aberle DR DeMello S Berg CD et al Results of the two incidence screenings in the National Lung Screening Trial N Engl J Med 369 920 931 2013 24004119 10.1056/NEJMoa1208962 PMC4307922

[25] Bekelman JE Lu H Pugh S et al Pragmatic randomised clinical trial of proton versus photon therapy for patients with non-metastatic breast cancer: The Radiotherapy Comparative Effectiveness (RadComp) Consortium trial protocol BMJ Open 9 e025556 2019 10.1136/bmjopen-2018-025556 PMC6797426 31619413

[26] Kalpathy-Cramer J, Napel S, Goldgof D, et al: Multi-site collection of lung CT data with nodule segmentations (QIN Lung CT Segmentation Challenge). The Cancer Imaging Archive, 2015. 10.7937/K9/TCIA.2015.1BUVFJR7

[27] Reference deleted.

[28] Reference deleted.

[29] Bloch N, Madabhushi A, Huisman H, et al: NCI-ISBI 2013 challenge: Automated segmentation of prostate structures. The Cancer Imaging Archive 370, 2015. 10.7937/K9/TCIA.2015.zF0vlOPv

[30] Reference deleted.

[31] Song M, Zhang C, Haihong E: An auto scaling system for API gateway based on Kubernetes. IEEE 9th International Conference on Software Engineering and Service Science. IEEE, Beijing, China, November 23-25, 2018, pp 109-112

[32] Elzein NM Majid MA Hashem IAT et al Managing big RDF data in clouds: Challenges, opportunities, and solutions Sustain Cities Soc 39 375 386 2018

[33] Yadav S: Comparative study on open source software for cloud computing platform: Eucalyptus, openstack and opennebula. Int J Eng Sci (Ghaziabad) 3:51-54, 2013

[34] Colen R Foster I Gatenby R et al NCI workshop report: Clinical and computational requirements for correlating imaging phenotypes with genomics signatures Transl Oncol 7 556 569 2014 25389451 10.1016/j.tranon.2014.07.007 PMC4225695

[35] Gevaert O Xu J Hoang CD et al Non-small cell lung cancer: Identifying prognostic imaging biomarkers by leveraging public gene expression microarray data—Methods and preliminary results Radiology 264 387 396 2012 22723499 10.1148/radiol.12111607 PMC3401348

[36] Velazquez ER Parmar C Jermoumi M et al Volumetric CT-based segmentation of NSCLC using 3D-Slicer Sci Rep 3 3529 2013 24346241 10.1038/srep03529 PMC3866632

[37] Grove O Berglund AE Schabath MB et al Quantitative computed tomographic descriptors associate tumor shape complexity and intratumor heterogeneity with prognosis in lung adenocarcinoma PLoS One 10 e0118261 2015 25739030 10.1371/journal.pone.0118261 PMC4349806

[38] Parmar C Grossmann P Bussink J et al Machine learning methods for quantitative radiomic biomarkers Sci Rep 5 13087 2015 26278466 10.1038/srep13087 PMC4538374

[39] Gurcan MN Pan T Shimada H et al Image analysis for neuroblastoma classification: Segmentation of cell nuclei Conf Proc IEEE Eng Med Biol Soc 2006 4844 4847 2006 17947119 10.1109/IEMBS.2006.260837

[40] Sertel O Kong J Shimada H et al Computer-aided prognosis of neuroblastoma on whole-slide images: Classification of stromal development Pattern Recognit 42 1093 1103 2009 20161324 10.1016/j.patcog.2008.08.027 PMC2678741

[41] Foran DJ Yang L Chen W et al ImageMiner: A software system for comparative analysis of tissue microarrays using content-based image retrieval, high-performance computing, and grid technology J Am Med Inform Assoc 18 403 415 2011 21606133 10.1136/amiajnl-2011-000170 PMC3128405

[42] Buckler AJ Bresolin L Dunnick NR et al Quantitative imaging test approval and biomarker qualification: Interrelated but distinct activities Radiology 259 875 884 2011 21325035 10.1148/radiol.10100800 PMC5410955

[43] Gillies R Radiomics: Informing cancer heterogeneity J Nucl Med 54 31 2013

[44] Kong J, Cooper L, Sharma A, et al: Texture based image recognition in microscopy images of diffuse gliomas with multi-class gentle boosting mechanism. 2010 IEEE International Conference on Acoustics, Speech and Signal Processing, IEEE, Dallas, TX, March 14-19, 2010, pp 457-460

[45] Saltz J, Almeida J, Gao Y, et al: Towards Generation, Management, and Exploration of Combined Radiomics and Pathomics Datasets for Cancer Research. Presented at the AMIA 2017 Joint Summits on Translational Science, San Francisco, CA, March 27-30, 2017 PMC5543366 28815113

[46] Fuchs TJ Buhmann JM Computational pathology: Challenges and promises for tissue analysis Comput Med Imaging Graph 35 515 530 2011 21481567 10.1016/j.compmedimag.2011.02.006

[47] Ghaznavi F Evans A Madabhushi A et al Digital imaging in pathology: Whole-slide imaging and beyond Annu Rev Pathol 8 331 359 2013 23157334 10.1146/annurev-pathol-011811-120902

[48] Klemm J, Basu A, Fore I, et al: The caBIG life sciences distribution, in: Ochs M, Casagrande J, Davuluri R (eds): Biomedical Informatics for Cancer Research. Boston, MA, Springer 2010, pp 253-266

[49] Genereaux BW Dennison DK Ho K et al DICOMweb: Background and application of the web standard for medical imaging J Digit Imaging 31 321 326 2018 29748852 10.1007/s10278-018-0073-z PMC5959831

[50] Saltz J Gupta R Hou L et al Spatial organization and molecular correlation of tumor-infiltrating lymphocytes using deep learning on pathology images Cell Rep 23 181 193.e7 2018 29617659 10.1016/j.celrep.2018.03.086 PMC5943714

[51] Keycloak Open Source Identity and Access Management. https://www.keycloak.org/

[52] Saltz J Sharma A Iyer G et al A containerized software system for generation, management, and exploration of features from whole slide tissue images Cancer Res 77 e79 e82 2017 29092946 10.1158/0008-5472.CAN-17-0316 PMC5987533

[53] Wilkinson MD, Dumontier M, Aalbersberg IJ, et al: The FAIR guiding principles for scientific data management and stewardship. Sci Data 3:160018, 2016 [Erratum: Sci Data 6:6, 2019] 10.1038/sdata.2016.18 PMC4792175 26978244

[54] National Cancer Institute Enhanced Data Sharing Working Group Recommendation: The Cancer Data Ecosystem Rockville, MD National Cancer Institute 2016

[55] Hammouda I, Knauss E, Costantini L: Continuous API design for software ecosystems. 2015 IEEE/ACM 2nd International Workshop on Rapid Continuous Software Engineering, IEEE, Florence, Italy, May 23-25, 2015, pp 30-33

[56] Bona JP, Nolan TS, Brochhausen M: Ontology-enhanced representations of non-image data in The Cancer Imaging Archive. Proceedings of the 9th International Conference on Biological Ontology (ICBO 2018), Corvallis, OR, August 7-10, 2018 PMC12076581 40371008

[57] Smith B Ashburner M Rosse C et al The OBO Foundry: Coordinated evolution of ontologies to support biomedical data integration Nat Biotechnol 25 1251 1255 2007 17989687 10.1038/nbt1346 PMC2814061

[58] Arp R Smith B Spear AD Building ontologies with basic formal ontology Cambridge, MA MIT Press 2015

[59] Schriml LM Arze C Nadendla S et al Disease ontology: A backbone for disease semantic integration Nucleic Acids Res 40 D940 D946 2012 22080554 10.1093/nar/gkr972 PMC3245088

[60] Bandrowski A Brinkman R Brochhausen M et al The ontology for biomedical investigations PLoS One 11 e0154556 2016 27128319 10.1371/journal.pone.0154556 PMC4851331

[61] Mungall CJ Torniai C Gkoutos GV et al Uberon, an integrative multi-species anatomy ontology Genome Biol 13 R5 2012 22293552 10.1186/gb-2012-13-1-r5 PMC3334586

[62] Kemp AS Bona J Nolan TS et al Semantic representations of multi-modal data, neuroinformatic processing pipelines, and derived results in the Arkansas Research Image Enterprise System (ARIES).Presented at theAmerican Medical Informatics Association (AMIA) Annual Symposium Washington DC November 16-20, 2019

[63] Urban T Ziegler E Lewis R et al LesionTracker: Extensible open-source zero-footprint web viewer for cancer imaging research and clinical trials Cancer Res 77 e119 e122 2017 29092955 10.1158/0008-5472.CAN-17-0334 PMC5679226

[64] Sharma A Tobler J CPTAC Pathology Portal Little Rock, AR The Cancer Imaging Archive 2020

[65] Iyer G, DuttaDuwarah S, Sharma A: DataScope: Interactive visual exploratory dashboards for large multidimensional data. 2017 IEEE Workshop on Visual Analytics in Healthcare (VAHC), IEEE, Phoenix, AZ, October 1, 2017, pp 17-23

[66] Russell P Fountain K Wolverton D et al TCIApathfinder: An R client for the cancer imaging archive REST API Cancer Res 78 4424 4426 2018 29871933 10.1158/0008-5472.CAN-18-0678

[67] Kikinis R, Pieper SD, Vosburgh KG: 3D Slicer: A platform for subject-specific image analysis, visualization, and clinical support, in: Jolesz FA (ed): Intraoperative Imaging and Image-Guided Therapy. New York, NY, Springer, 2014, pp 277-289

[68] Perez F, Granger BE: Project Jupyter: Computational narratives as the engine of collaborative data science. https://blog.jupyter.org/project-jupyter-computational-narratives-as-the-engine-of-collaborative-data-science-2b5fb94c3c58

[69] Moore SM Maffitt DR Smith KE et al De-identification of medical images with retention of scientific research value Radiographics 35 727 735 2015 25969931 10.1148/rg.2015140244 PMC4450976

[70] Prior F Almeida J Kathiravelu P et al Open access image repositories: High-quality data to enable machine learning research Clin Radiol 75 7 12 2020 31040006 10.1016/j.crad.2019.04.002 PMC6815686

[71] Bennett W Smith K Jarosz Q et al Reengineering workflow for curation of DICOM datasets J Digit Imaging 31 783 791 2018 29907888 10.1007/s10278-018-0097-4 PMC6261183

[72] Crosas M The Dataverse Network: An open-source application for sharing, discovering and preserving data Dlib Mag 17 2 2011

[73] Brase J: DataCite-A global registration agency for research data. 2009 Fourth International Conference on Cooperation and Promotion of Information Resources in Science and Technology. IEEE, Beijing, China, November 21-23, 2009, pp 257-261

[74] Hightower K, Burns B, Beda J: Kubernetes: Up and running: Dive into the future of infrastructure. Sebastopol, CA, O’Reilly Media, 2017

[75] GitHub: UAMS/DBMI PosdaTools. https://github.com/UAMS-DBMI/PosdaTools

[76] GitHub: SBU-BMI quip_distro. https://github.com/SBU-BMI/quip_distro

[77] GitHub: caMicroscope. https://github.com/camicroscope/caMicroscope

[78] GitHub: Kong. https://github.com/Kong/kong

